# Equine Infectious Anemia Virus Cellular Partners Along the Viral Cycle

**DOI:** 10.3390/v17010005

**Published:** 2024-12-24

**Authors:** Cécile Schimmich, Astrid Vabret, Stéphan Zientara, José Carlos Valle-Casuso

**Affiliations:** 1 ANSES Animal Health Laboratory, PhEED Unit, 14430 Goustranville, France; cecile.schimmich.ext@anses.fr; 2 Department of Virology, University of Caen Normandy, Dynamicure INSERM UMR 1311, Centre Hospitalo Universitaire (CHU) Caen, 14000 Caen, France; vabret-a@chu-caen.fr; 3 UMR VIROLOGIE, INRAE, École Nationale Vétérinaire d’Alfort, ANSES Laboratoire de Santé Animale, Université Paris-Est, 94700 Maisons-Alfort, France; stephan.zientara@anses.fr; 4 Mixed Technological Unit “Equine Health and Welfare—Organisation and Traceability of the Equine Industry” (UMT SABOT), 14430 Goustranville, France

**Keywords:** EIAV, HIV-1, host–pathogen interactions, restriction factors, *lentivirus*, viral cycle

## Abstract

Equine infectious anemia virus (EIAV) is the simplest described *lentivirus* within the *Retroviridae* family, related to the human immunodeficiency viruses (HIV-1 and HIV-2). There is an important interplay between host cells and viruses. Viruses need to hijack cellular proteins for their viral cycle completion and some cellular proteins are antiviral agents interfering with viral replication. HIV cellular partners have been extensively studied and described, with a special attention to host proteins able to inhibit specific steps of the viral cycle, called restriction factors. Viruses develop countermeasures against these restriction factors. Here, we aim to describe host cellular protein partners of EIAV viral replication, being proviral or antiviral. A comprehensive vision of the interactions between the virus and specific host’s proteins can help with the discovery of new targets for the design of therapeutics. Studies performed on HIV-1 can provide insights into the functioning of EIAV, as well as differences, as both types of virus research can benefit from each other.

## 1. Introduction

Equine infectious anemia virus (EIAV) is the causative agent of equine infectious anemia (EIA), an almost worldwide disease affecting equids such as horses and donkeys. EIAV belongs to the *Retroviridae* family, *lentivirus* genus. Lentiviruses cause slow chronic diseases to their specific host. The two main differences between HIV-1 and EIAV regard the transmission and the disease. EIAV spreads among herds through blood-feeding insects such as horse flies (*Tabanidae*) or stable flies (*Stomoxys*) [1] and can also be transmitted through unsanitary veterinary practices, with the use of contaminated needles for example. The disease caused by EIAV infection is characterized by three clinical phases: acute, chronic and finally asymptomatic [2,3], and the fact that it does not cause a degenerative immunodeficiency. Within the first weeks, the infected equid undergoes an acute phase with clinical signs such as thrombocytopenia, fever, weight loss and depression. Then, settles a chronic phase with alternating viremia that overlaps with clinical signs. In general, some equids progress to an asymptomatic phase, without any visible signs of the infection, except for the presence of circulating antibodies against EIAV. During this last phase, the infected horses remain carriers of the virus for the rest of their lives, as an integrated and silent provirus, which can be reactivated by stresses or immune suppression [4,5].

Recent work reviewed known EIAV restriction factors [6]. Restriction factors can be triggered by immune sensing, interferon induced or not. Viruses, such as HIV-1, acquire countermeasure against these host restriction factors, often as multi-purpose accessory proteins. What is contrasting between EIAV and HIV-1 is that HIV-1 harbors more accessory proteins, such as Tat, Rev, Nef, Vif, Vpu and Vpr, and only Tat, Rev and S2 for EIAV, the simplest described *lentivirus*. In this review, we focus on the described EIAV interacting host factors, both restriction factors and viral co-factors, as viral cycle completion necessitates an interplay between viral and cellular proteins. We describe steps from cell entry to proviral genome integration and budding of new virions. We take advantage of HIV-1 research, as well as other lentiviruses, to better describe the mechanisms and give a comprehensive landscape of EIAV infection cycle and involved host factors.

## 2. EIAV Viral Cycle

### 2.1. The Virus

**Figure 1:**
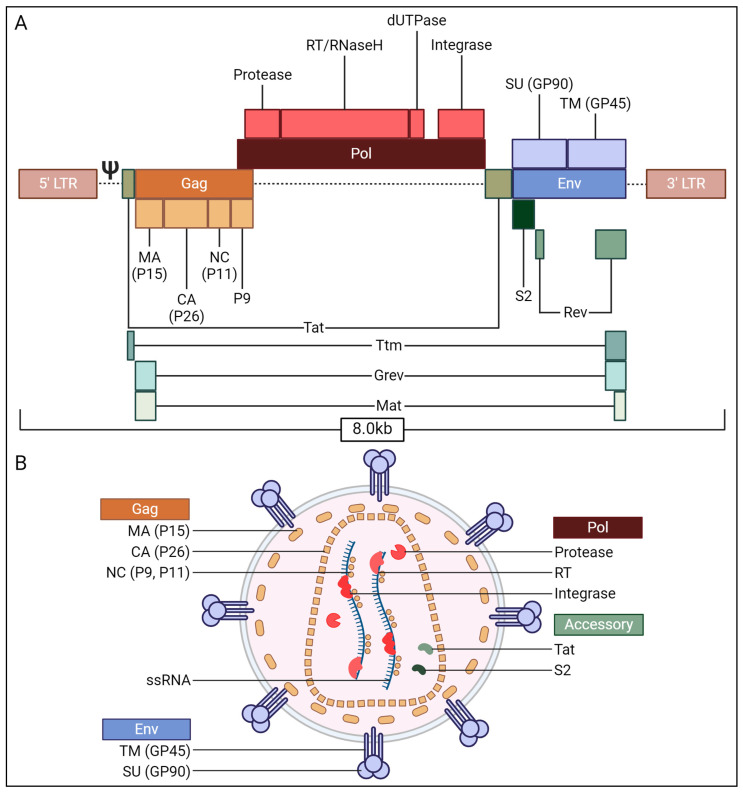
EIAV proviral genome structure (**A**) and assembled virion organization (**B**) CA: capsid; Grev: Gag–Rev fusion; LTR: long terminal repeat; MA: matrix; Mat: Matrix–TM fusion; NC: nucleocapsid; Rev: regulator of expression of viral proteins; RNaseH: ribonuclease H; RT: reverse transcriptase; SU: surface protein; Tat: trans-activator of transcription; TM: transmembrane protein; Ttm: Tat–TM fusion.

The EIAV genome is about 8.0 kb ssRNA, as two identical copies within every viral particle (Figure 1), composed by the stereotyped lentiviral open reading frames: *gag* (group specific antigen), *pol* (polymerase), *env* (envelope) and three accessory proteins, Tat (trans-activator of transcription), Rev (regulator of expression of viral proteins) and S2 [7]. The Gag polyprotein is cleaved into four structural proteins: nucleocapsid (p11 and p9), capsid (p26) and matrix (p15). The Pol polyprotein is cleaved into the enzyme proteins: protease, integrase and reverse transcriptase/ribonuclease H (RT/RNAseH). The Env polyprotein is processed into a transmembrane domain protein (gp45) and a surface domain protein (gp90). Finally, regarding accessory proteins, EIAV possesses Tat and Rev which have similar activities like Tat and Rev from HIV-1. Tat is required for transcription of the viral RNA. Rev allows for the nuclear export of unconventional transcripts. This includes unspliced and partially spliced viral RNA transcripts, necessary for some viral proteins translation and genomic RNA that will be encapsidated in newly formed virions. S2 has no known structural or functional homologous proteins, and its expression is not required for *in vitro* viral replication [8] but is necessary for acute disease in vivo [9,10]. Remarkably, other lentiviruses such as HIV-1 encode in their genome accessory proteins like Vif (viral infectivity factor), Nef (negative regulatory factor), Vpr (viral protein r) or Vpu (viral protein u) [11]. Other EIAV accessory proteins have been briefly described: Ttm (Tat–transmembrane protein fusion) [12], Mat (Matrix–transmembrane protein fusion) and Grev (Gag–Rev fusion) more recently [13,14]. Ttm, identified in a single study [12], is a fusion protein formed from Tat and the C-terminal domain of the transmembrane envelope protein, found in acutely infected horses with pathogenic EIAV. However, no functional characterization has been conducted since. Recently, Mat mRNA was discovered, overlapping the matrix coding region of gag and the 3′ terminal part of the transmembrane envelope protein coding region. Rev facilitates the export of Mat mRNAs from the nucleus via a newly identified Rev response element (RRE), RRE-2, located differently from the classic RRE [13]. Lastly, Grev, similar to Rev, was described as specifically mediating the expression of Mat, possibly with Rev’s assistance, but lacking other Rev functions. Grev exports Mat mRNAs through the CRM1 pathway [14].

### 2.2. The Cycle

**Figure 2:**
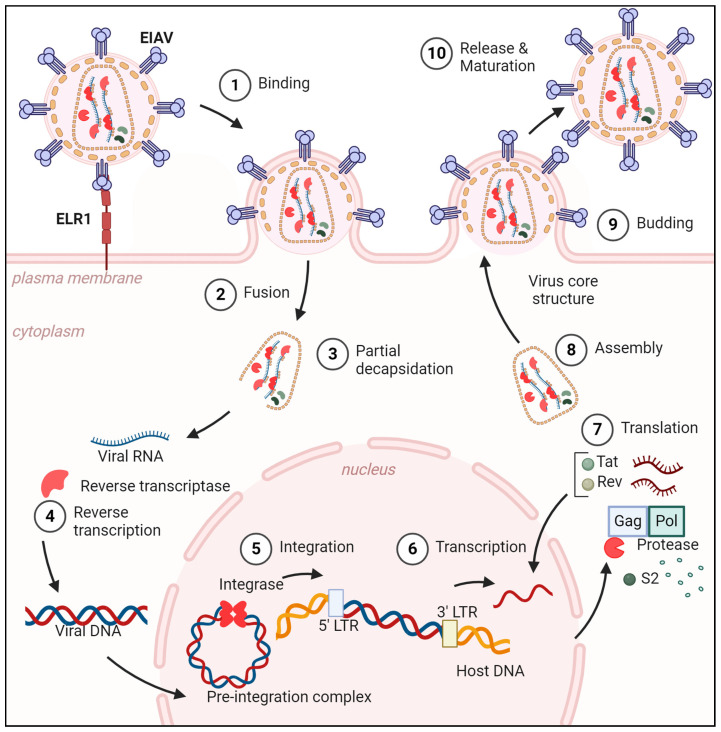
EIAV infection cycle. ELR1: equine *lentivirus* receptor 1; LTR: long terminal repeat.

The EIAV viral cycle (Figure 2) is thought to be similar as the HIV-1 cycle, which is much more documented. It begins with the entry into target equine cells possibly via a single receptor, in contrast to other lentiviruses that use co-receptors [15]. One of the earliest steps after entry of the retroviral cycle is the reverse transcription of the RNA genome into DNA. For a long time, reverse transcription and uncoating were thought to take place in the cytoplasm before nuclear import. More recently, it appears that blockage of the nuclear pores impairs reverse transcription and uncoating, implying that both steps happen inside the nucleus [16]. These two steps may not be strictly compartmentalized to the cytoplasm or the nucleus and a mix of both may occur [17]. Retroviral infection requires viral genome integration into the host genome as an essential part of viral cycle. But, unlike other retroviruses, like murine leukemia virus (MLV), often used as a comparison, lentiviruses have the capacity to enter the nucleus independently of the cell cycle, meaning they can infect non-dividing cells [18]. The viral integrase binds to the newly formed viral DNA as well as other cellular and viral proteins to form the pre-integration complexes (PICs), which allow for the integration of the provirus into the host DNA [19]. Integration into the host genome has several consequences. It allows for a persistent infection of the host, and the provirus can be passed onto daughter cells following cell division. Integrated provirus impacts the transcription of cellular genes near the integration site [19,20]. Then, viral RNA can be transcribed. Viral mRNA can be completely or partially spliced to produce the viral proteins and full genomic RNA to be encapsidated in the newly produced virions. Here, accessory proteins Tat and Rev are key actors. Tat allows for the transcription of the provirus and Rev for the export of unspliced or partially spliced mRNAs, both with the help of endogenous proteins. The new viral particles are formed with newly translated viral proteins and full viral RNA genome. It is the viral protease that proteolyzes the structural proteins and enzymatic proteins from two long polypeptides Gag and Gag-Pol [21,22]. Again, viral assembly and budding require viral and cellular proteins interplay, but the host also develops an anti-viral response against the infection. EIAV or HIV-1, more extensively described, develop countermeasures against cellular antiviral response proteins known as restriction factors. These act upstream innate immunity as they do not require viral sensing immune response [23].

In vivo, EIAV is a macrophage-tropic *lentivirus* [24]. Blood monocytes are permissive for EIAV infection, but replication is upregulated in mature macrophages [25]. Infection of macrophages allows for the establishment of a viral reservoir, as well as a mean to disseminate the virus to macrophages-rich organs such as spleen, liver and kidney, described as the main sites of virus replication in infected equids [26].

## 3. Host Factors Involved in the Early Phase of EIAV Infection Before Integration

### 3.1. Entry

The receptor for EIAV in equine cells was described in 2005 as a protein from the family of tumor necrosis factor receptor (TNFR), named equine *lentivirus* receptor 1 (ELR1) [15]. To enter the cell, the EIAV envelop protein surface domain (gp90) binds to the receptor. It appears that non-permissive cells to EIAV infection could be infected upon transduction of ELR1, indicating that the entry of the virus may be mediated by this single receptor [15]. The entry of EIAV is mediated by this receptor and utilizes a pH-dependent clathrin-mediated endocytic pathway [27,28,29]. Interestingly, EIAV is the only *lentivirus* that requires only one receptor, without a co-receptor, which is the case for other retroviruses such as MLV. Furthermore, ELR1 is part of the TNFR family, like one of the receptor of the feline immunodeficiency virus (FIV), CD134 [30,31]. The C-terminal part of ELR1, the CRD1 (cysteine-rich domain 1) segment, is the critical domain that allows for gp90 binding for the viral entry, and leucine in position 70 appears to be the critical residue within CRD1 for the interaction [32].

### 3.2. Fate of the Capsid

While the receptor is the only cellular factor described helping the early steps of EIAV infection as of today, several restriction factors preventing infection have been studied in the following cycle steps. For example, restriction factor 1 (Ref1) is a species-specific restriction factor studied and described for EIAV. It is a human gene that blocks N-tropic MLV infection, related to the murine gene Friend virus susceptibility factor 1 (Fv1) that restricts N-tropic or B-tropic MLV infection. Fv1 protein is homologous to endogenous retroviral Gag protein [33]. Potentially related to Fv1, *lentivirus* susceptibility factor (LV1) is found in some non-human primate cell lines and displays similar action of restricting HIV-1 and SIVmac. EIAV is unable to infect both human and African green monkey (AGM) cell lines [34]. It appears that Ref1/Fv1/Lv1 are species-specific variants of TRIM5α, as they can confer resistance to N-tropic MLV infection in non-restricting cell lines [35]. Later, it was shown that the murine gene FV1 had a restriction effect on the infection by EIAV, most likely by the same mechanism as MLV restriction: blocking the viral DNA import to the nucleus by capsid binding, after the reverse transcription step [36].

The tripartite motif-containing protein 5, also known as RING finger protein 88 (TRIM5α), is a retrovirus restriction factor with a species-specific activity, that protects cells from cross-species infections with retroviruses. A specific retrovirus from one species will be restricted by the cellular TRIM5α from another species. It blocks the viral cycle before integration, by binding to the assembled capsid, not the soluble forms, preventing the uncoating [37]. EIAV replication is impaired by TRIM5α, marginally by TRIM34, a TRIM protein closely related to TRIM5 [38,39]. It appears that rhesus monkey TRIM5α (TRIM5α_rh_) restricts even more potently HIV-1 as well as EIAV than human TRIM5α, particularly thanks to the variable region 1 of the TRIM5α_rh_ B30.2 domain [40].

Myxovirus resistance (MX) proteins are restriction factors from the family of dynamin-like GTPases. Their expression is stimulated by type I (alpha beta) and type III (lambda) interferon, triggered by viral infection. Human myxovirus resistance protein B (hMXB) restricts HIV-1, but not EIAV [41]. In contrast, equine MX2 (eMX2) restricts EIAV, as well as other lentiviruses (HIV-1, HIV-2, various simian immunodeficiency viruses, SIVs) and the retrovirus MLV. Similar to hMXB, the amount of 2-LTR circular HIV-1 DNA was reduced by eMX2, indicating that eMX2 most likely blocks the virus prior to nuclear entry [41]. The N-terminal part of eMX2 is essential for capsid binding, and it appears that, upon viral infection, eMX2 is distributed around the nucleus. Its localization and ability to bind lentiviral capsids, including EIAV’s, allows eMX2 to block the nuclear entry of proviral DNAs and therefore has antiviral abilities [42].

### 3.3. Reverse Transcription

The reverse transcription is a key step of lentiviral infections. Sterile alpha motif and histidine/aspartic domain-containing protein 1 (SAMHD1) is described as a restriction factor expressed in myeloid cells and can inhibit HIV-1 infection interfering with reverse transcription as it depletes the nucleotide pool of the cell. It is composed of sterile alpha motif (SAM) and histidine-aspartic (HD) domains. It appears that only the HD domain is required for restriction of HIV-1 and other lentiviruses, including EIAV. This restriction factor only partially blocks EIAV infection, meaning that EIAV could have a mechanism to counteract it [43]. Indeed, EIAV can infect macrophages with functional SAMHD1. First, it was observed that EIAV and other non-primate lentiviruses (FIV, bovine immunodeficiency virus, BIV) fail to proteosomally degrade SAMHD1 through their accessory proteins, Vpr or Vpx, a feature allowing this degradation uniquely gained by primate lentiviruses [44]. It was then demonstrated that, during EIAV infection, Rev downregulates SAMHD1 through the lysosomal pathway. Rev hijacks beclin 1 (BECN1) and phosphatidylinositol 3-kinase catalytic subunit type 3 (PIK3C3) to mediate SAMHD1 degradation in a canonical macroautophagy/autophagy-independent pathway, giving some explanation elements to EIAV’s ability to infect SAMHD1-functional equine macrophages [45].

Apolipoprotein B mRNA editing enzyme-catalytic polypeptide 3 or APOBEC3 (A3) proteins are known potent inhibitors of retroviruses and retrotransposons, by inducing hypermutation in DNA, counteracted by HIV-1 viral protein Vif [46,47]. The same goes with FIV Vif protein; it counteracts feline A3 proteins [48,49]. EIAV is the only *lentivirus* that does not encode a Vif protein [50]. The equine genome encodes six different A3 proteins, four containing one consensus active site of A3, cytidine deaminase (CDA), and two containing two CDA sites. Equine A3 (eA3) mRNA are found in equine cells and have an inhibitory effect on Vif defective HIV-1. Equine A3Z3 and A3Z2c-Z2d are potent inhibitors of EIAV, while other versions have a weak inhibitory effect. On the other hand, human A3 can inhibit EIAV. Human and equine A3 can bind HIV-1 and EIAV nucleocapsid proteins and be incorporated into virions. It appears that EIAV can counteract equine A3’s effect after virion incorporation [51]. Furthermore, as EIAV lacks a Vif protein, it was proposed that EIAV can resist A3 antiviral activity by different strategies: tolerating the incorporation of A3 proteins that are the least active against EIAV (A3Z1b, A3Z2a-Z2b and A3Z2e) and evolve its macrophage tropism as they show very low levels of A3 expression [52].

## 4. Host Factors Involved in the Nuclear Import of EIAV

For lentiviruses, the import of lentiviral DNA to the nucleus in non-dividing cells is a necessary step in the viral cycle. Access to the nucleus without disrupting the nuclear envelop requires the help of cellular proteins. Nucleoporins (NUPs) form a family of proteins that constitute the nuclear pore complex (NPC), regulating the flow in and out the nucleus via nuclear pores. It was described that the HIV-1 capsid N-terminal part can bind to the NUP153 C-terminal part, which is important for the PIC nuclear import via the NPC. As this interaction is conserved for EIAV, it appears that the Ca–NUP153 interaction has the same role for EIAV infectivity [53].

Along with NUPs, transportin-3 (TNPO3) is another actor of lentiviral nuclear import, as a receptor for serine/arginine-rich (SR) proteins. Contrary to what was previously thought, TNPO3 is required during HIV-1 infection for nuclear import via capsid binding rather than integrase interaction [54]. For lentiviral nuclear import, TNPO3 was described as necessary, and specifically the C-terminal part of the protein, which is a cargo binding site [55]. Finally, TNPO3 was shown to be required for nuclear import but not necessary for integration, which correlates with the fact that it binds capsid protein and not integrase. A knockdown of TNPO3 decreased EIAV infection, as well as HIV-1, SIVmac, HIV-2 and BIV [56].

## 5. Inside the Nucleus: Host Factors Involved in the Integration of EIAV’s Provirus into the Host’s Genome and Proviral Transcription

### 5.1. Integration

Once in the nucleus, the PIC needs to be integrated into the host’s own DNA as a provirus. Then, viral transcription can occur to produce new viral proteins after translation, as well as the viral complete genomic RNA. A key viral protein in this step is the integrase. It is packaged within every virion and is also imported to the nucleus as part of the PIC to catalyze the proviral integration reaction. Once again, host factors are also key players in this step. Lens epithelium-derived growth factor (LEDGF), also referred to as p75, guides lentiviral integration site selection. EIAV, like other lentiviruses, appears to integrate in active transcription units [57]. Furthermore, it appears that EIAV favors integration in A/T rich palindromic consensus sequences [58]. Interaction with LEDGF/p75 is limited to lentiviral integrases, including EIAV’s and not to all *Retroviridae* family’s integrases. A single amino acid appears to be key for the interaction for all lentiviruses integrases with LEDGF, as the mutation of an aspartic acid in position 366 to an asparagine in LEDGF impaired all integrase–LEDGF interactions [59].

### 5.2. Transcription

After integration of the proviral DNA, EIAV transcription requires cellular co-factors. Tat protein is a key player here, as it will transactivate the transcription of viral transcripts. EIAV Tat protein, like HIV-1 and HIV-2 Tat proteins, binds the protein named Tat-associated kinase (TAK) through activation domains of Tat. TAK is sensitive to dichloro-1-beta-D-ribofuranosylbenzimidazole, a nucleoside analogue known for inhibiting a limited number of kinases and known to inhibit Tat transactivation. The C-terminal part of the large subunit of RNA polymerase II is a substrate of TAK, and its phosphorylation triggers elongation of transcription. Altogether, TAK appears to be a key cell protein for EIAV viral transcription [60].

During EIAV and HIV-1 infections, the accessory protein Tat stimulates in trans LTR-directed gene expression. Tat is required to increase the transcription of viral mRNAs, through the binding to the TAR (Tat-responsive element), located at the 5′ end of all viral transcripts [61]. For EIAV, the equine cyclin T1 supports the binding of Tat to the TAR and the resulting transactivation. For HIV-1, it was described that Tat requires the human cyclin T1 in order to bind with the TAR. Although human cyclin T1 can bind EIAV Tat protein, it does not allow for transactivation. A single amino acid at position 29 in the human or equine cyclin T1 is responsible for this observation. An exchange of a valine to a leucine at position 29 in the human cyclin T1 allows for EIAV transactivation, whereas this switch from leucine to valine in equine cyclin T1 makes it inactive for Tat interaction with TAR [62]. Cyclin T1 associated with CDK9 kinase are part of positive transcription elongation factor (P-TEFb). Equine P-TEFb is remarkable as it binds Tat, TAR and allows for the transcription of EIAV [63]. PU.1 is a macrophage-specific transcription factor, encoded by the *SPI1* gene. It was demonstrated that, within EIAV LTRs, there are three PU.1 binding sites that are necessary for EIAV viral expression in primary macrophages. These sites are critical within LTRs for Tat transactivation but are not sufficient to promote EIAV replication in macrophages; there are other required elements. Another member of the TRIM protein family was reported to interact with EIAV Tat protein: TRIM32, also known as HT2A or C3HC4, binding specifically Tat activation domain within the nucleus. This interaction is shared between HIV-1, HIV-2 and EIAV Tat proteins and may promote Tat activity in vivo [64].

SR proteins are splicing factors required for constitutive and alternative mRNA splicing, known as cargo proteins of TNPO3. A purine rich exonic splicing enhancer (ESE) sequence was found in a bicistronic mRNA composed of four exons that encodes for EIAV Tat (exon 1 and 2) and Rev (exon 3 and 4). Rev itself interacts with this ESE in exon 3, to block SR protein-ESE binding, skipping exon 3. It appears that Rev downregulates its own expression through this interaction [65,66]. The SR protein binding to this ESE appears to be alternative splicing factor 1 (ASF1), also known as pre-mRNA-splicing factor SF2 (SF2). It particularly binds to a 55-nucleotide purine-rich sequence proximal to the 5′ end splice site of exon 3 [67,68]. Within the nucleus, Rev effector binding protein (REBP), a nuclear kinesin-like protein, can bind HIV-1 rev and EIAV Rev. It may mediate the nuclear export function of Rev [69].

## 6. Host Factors Involved in the Nuclear Export of EIAV’s Transcripts

Within the nucleus, after integration and transcription of viral DNA, the infection cycle faces a new challenge: exporting non-conventional RNAs. Indeed, the cell already takes care of the export to the cytoplasm of fully spliced cellular and viral RNAs through conventional RNA export mechanisms with the chromosomal maintenance 1 export protein (CRM1). During EIAV viral cycle, unspliced transcribed RNA, as its viral RNA directly packaged in newly formed virions as well as partially spliced RNAs are required. The accessory protein Rev is essential for the export of unspliced RNAs, but not sufficient and requires the support of cellular proteins.

Nucleoporins (NUPs) are proteins composing the nuclear pore complex (NPC) and handling import and export to the nucleus. NUP98, a member of NUPs, directly interacts with EIAV Rev nuclear export signal (NES), determined by yeast-two hybrid assay. This is a conserved interaction between EIAV Rev and HIV-1 Rev, shown to have a role in modulating the Rev-dependent RNA export during HIV-1 infection [70]. Although part of the RRE (Rev Responsive Element) mediated RNA export, Nup98 is not sufficient to export RNAs, as shown by a Nup98–Rev fusion, in contrast to a CRM1–Rev fusion protein that mediates the efficient nuclear export of RRE RNAs.

## 7. Host Factor Gatekeeper at the Endoplasmic Reticulum

After exiting the nucleus for the cytoplasm, some viral RNAs are translated and go through the endoplasmic reticulum. Viperin (Virus inhibitory protein, endoplasmic reticulum-associated, interferon inducible, also known as RSAD2) is a multifunctional protein, characterized by a broad antiviral activity. Equine viperin has antiretroviral activities against EIAV at three different levels. First, it inhibits Gag and Env budding; secondly, it reduces membrane targeting protein synthesis in the endoplasmic reticulum (ER), namely Env and ELR1; and lastly, it impairs protein trafficking, notably ELR1, EIAV’s receptor. The alpha-helix domain of equine viperin is required for the budding of EIAV Gag and the release of Env and ELR1, as well as the structural change in ER which is the main mechanism of viperin action. It has no effect on the production of EIAV accessory proteins Tat and Rev. The counteracting effect of EIAV was not reported against equine viperin, as even though EIAV infection upregulates viperin expression, physiological levels of equine viperin are relatively low and may not be sufficient to restrict EIAV [71]. Another described antiviral mechanism of action of viperin is through the conversion of cytidine triphosphate to 3′-deoxy3′,4′-didehydro-CTP. This change results in chain termination for RNA-dependent RNA polymerase for Flaviviruses, inhibiting the viral replication. A similar mechanism could have a role in the antiviral effect of viperin for EIAV [72].

Schlafen (*Slfn*) genes are part of the interferon stimulated genes (ISGs), directly induced by viral infection sensing. It was first shown that SLFN11 protein had an antiviral activity against HIV-1, by impairing viral protein synthesis and binding to transfer RNA [73]. The equine version of this protein, equine SLFN11 has the same effect against EIAV [74]. Indeed, equine SLFN11 seems to impair EIAV mRNA translation with a similar mechanism that the human version does with HIV-1.

## 8. Host Factors Preventing EIAV Assembly and Budding

### 8.1. Assembly

The next steps of the viral cycle include assembly and budding of the newly formed virions.

Recently, CCHC-type zinc-finger-containing protein 3 (ZCCHC3) was identified as having an antiviral activity against HIV-1, as well as against other retroviruses including EIAV [75]. This host protein has a dual antiviral mechanism. It binds to Gag nucleocapsid protein, being packaged inside new virions and making them defective. It also sequesters viral RNA in P-bodies in the cytoplasm by binding the viral LTR, impairing viral translation. Overall, ZCCHC3 decreases viral production and infectivity. Interestingly, this restriction factor is not directly antagonized by any viral accessory proteins.

### 8.2. Budding

Tetherin protein has shown antiviral properties, by physically tethering new virions to the cell plasma membrane. It is induced by interferon production. Equine type I interferon (IFN-alpha1) shares a similar mechanism with human IFN-alpha1, inducing tetherin gene expression, triggered by viral infection and inhibiting virion release from the plasma membrane [76]. For EIAV, Env antagonizes the equine tetherin and, for HIV-1, it is Vpu that antagonizes human tetherin. These counteracting mechanisms are virus and species-specific: the antiviral activity of equine tetherin is counteracted by EIAV Env, but not HIV-1 Vpu, as well as that of human tetherin which is only counteracted by HIV-1 Vpu and not EIAV Env [77]. However, interestingly, it appears that feline tetherin restricts FIV as well as HIV-1, impairing the budding of new virions but not preventing cell-to-cell spread of the infection [78]. It was investigated whether horse and donkey tetherin could induce different responses, as both species can be infected by EIAV, with different clinical response. Both orthologs have similar antiviral responses against EIAV infection and induction of NF-κB signaling, meaning that the different clinical responses cannot be explained by tetherin differences [79]. Finally, the localization of tetherin at the plasma membrane is important for it to complete its physical antiviral role. It was suggested that the N-glycosylation of equine tetherin allows for the regulation of its localization. Without the N-glycosylation, it colocalized to endosomal compartments of the cells, by affecting anterograde trafficking of the protein, not able to tether virions [80].

The endosomal sorting complex required for transport (ESCRT) machinery, comprising several cytosolic proteins and associated proteins, is an actor of viral budding. This key step in the viral cycle is mainly driven by Gag proteins. The interaction between EIAV late assembly domain and the cellular AP-2 (adaptor protein 2) clathrin-associated adapter protein complex was reported. EIAV utilizes the cellular AP-2 complex to accomplish virion assembly and release, thanks to a YXXL sequence on the p9 protein, binding specifically the AP-50 subunit of AP-2 [81]. Furthermore, p9 was described as an interactor of AIP1/ALIX (ALG-2 [apoptosis-linked-gene-2 product]-interacting protein X) as a link to ESCRT-III for viral budding. This same interaction was reported with HIV-1 p6, also within its late domain [82], but not for FIV [83]. Notably, the prototype YPDL-type late domain encoded by EIAV Gag acts by recruiting AIP-1/ALIX and the expression of a truncated form of AIP-1/ALIX or small interfering RNA-induced AIP-1/ALIX depletion specifically inhibits EIAV YPDL-type late domain function for viral release [84]. Interestingly, HIV-1 and EIAV’s late domain in Gag protein are different in sequence but have the same function and bind the same site on ALIX protein. Crystallography of late domains with ALIX revealed that they both bind the same ALIX sites but adopt different conformations. A key peptide sequence in the late domain is YPXnL in HIV-1 or EIAV [85]. With another study, the interaction between HIV-1 p6 or EIAV p9 and ALIX was further characterized. The docking site in ALIX for p6 or p9 is active when ALIX is associated with the plasma membrane, showing some regulation of the interaction event between the cell protein and viral protein for the budding process [86]. For retroviral budding, ALIX needs to interact with the ESCRT-III component of charged multivesicular body protein 4 (CHMP4), at the N-terminal Bro1 domain. As stated before, the cytoplasmic ALIX cannot interact with viral proteins or CHMP4. There is an interaction between Patch 2 in the Bro1 domain of CHMP4 the ALIX docking site, the tumor susceptibility gene 101 protein (TSG101), locking ALIX in a closed conformation. It is critical that this autoinhibitory interaction is relieved for ALIX to interact and help with viral budding [87].

Another protein family regulating protein trafficking within the cell are soluble N-ethylmaleimide-sensitive factor attachment protein receptor proteins (SNARE proteins). They catalyze the fusion of intracellular vesicles with the plasma membrane. It was demonstrated that the silencing of SNARE proteins resulted in a decrease in production of HIV-1 and EIAV virions. The role of N-ethylmaleimide-sensitive factor (NSF) was highlighted for that process, as this factor is required for the recycling of the SNARE complex. Indeed, with less cellular trafficking, Gag transport and addressing the plasma membrane is affected. Viral budding requires the interplay between Gag viral proteins and cellular trafficking proteins, all at the cell membrane at the right time [88].

Serine incorporators 3 and 5 (SERINC3, SERINC5) are proteins localized at the plasma membrane, having many transmembrane domains and impairing viral entry through fusion with the plasma membrane. They were shown to restrict HIV-1 infectivity and to be counteracted by HIV-1 Nef and MLV glycoGag [89]. EIAV S2 shares no sequence homology to any known proteins, to Nef or glycoGag, but it appears to counteract SERINC5 antiviral activity, just like HIV-1 Nef, by excluding SERINC5 from viral particles. SERINC5 is highly expressed in blood-derived cells. Also, EIAV S2 can rescue the phenotype of a Nef-defective HIV-1, by counteracting SERINC5 instead of Nef. S2 requires a predicted ExxxLL motif to recruit the clathrin adaptor AP-2 to counteract SERINC5, just like Nef. S2-mediated infectivity enhancement requires functional endocytic machinery as it is impaired by clathrin-mediated endocytosis inhibitors. Through the endocytosis machinery, S2 retargets SERINC5 to the late endosomal compartment, as well as promoting host factor degradation. EIAV S2 interacts with SERINC5 and downregulates it from the membrane via AP-2-mediated endocytosis [90]. Similarly to HIV-1 Nef and MLV glycoMA described mechanisms, S2 targets SERINC5 to lysosomal degradation, by a direct interaction relying on the myristoylation of S2 at the plasma membrane. Equine SERINC5 expression is much weaker than SERINC5 from other species, but it still shows strong antiviral activity [91]. Interestingly, different retroviruses developed similar mechanisms to antagonize the SERINC family of proteins, host factors that have an antiviral activity.

## 9. Other Cellular Interactors of EIAV Proteins

Some cellular proteins do not have a specific role in the viral cycle, but they can still interfere with it. Two mechanisms having an antiviral effect against EIAV were described through the interaction of EIAV Rev and two cellular factors [92]. Kelch-like ECH-associated protein 1 (Keap1) is an actor of innate immunity and has an antiviral activity by interacting with EIAV Rev in the cytoplasm. Keap1 normally sequestrates nuclear factor erythroid 2-related factor (Nrf2) in the cytoplasm. Usually, with oxidative stress, Nrf2 is phosphorylated, translocated to the nucleus and promotes antioxidant response element (ARE)-driven genes transcription, which have antiviral effects. EIAV Rev binds competitively to Keap1 instead of Nrf2. This has two consequences: Rev is sequestrated to the cytoplasm, being unable to translocate to the nucleus after being translated and allow for the nuclear export of viral RNAs, and Nrf2 is free to go to the nucleus and promote its target gene transcription. These two mechanisms have an antiviral effect against EIAV.

S2 was shown to be required for infectivity in vivo. Apart from that, some cellular partners like proteins OS-9 (osteosarcoma) and PSMC3 (proteasome 26S ATPase subunit 3) were discovered to interact with S2, without any associated function or mechanism as of today [93].

## 10. Conclusions

**Figure 3:**
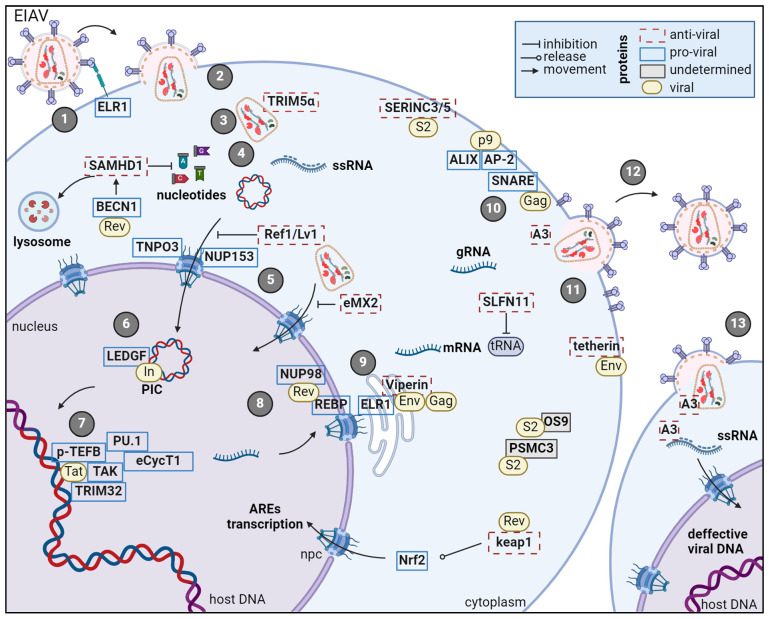
EIAV viral cycle and interacting host proteins. 1: Viral binding to its receptor; 2: Fusion; 3: Partial decapsidation; 4: Reverse transcription; 5: Nuclear import of viral DNA or viral capsid; 6: Integration of PIC into host DNA; 7: Transcription; 8: Nuclear export of full-length gRNA, fully spliced mRNA or partially spliced mRNA; 9: Translation; 10: Assembly of new virions; 11: Budding; 12: Release and maturation of newly formed virions; 13: Infection with defective virions leading to defective infection. ssRNA: single-strand RNA; mRNA: messenger RNA; gRNA: genomic RNA; tRNA: transfer RNA; rer: rough endoplasmic reticulum; AREs: antioxidant response elements; PIC: pre-integration complex.

In this review, we describe thirty known host cellular proteins having a role in EIAV infection (Figure 3, Table 1). EIAV developed the capacity to use seventeen cellular proteins to complete its viral cycle and the host cell developed infection countermeasures to block the viral cycle represented by eleven antiviral cellular proteins. Two cellular proteins were described as interacting with EIAV S2, without any described functions, PSMC3 and OS-9. No role of these two cellular proteins is already described for other retroviruses. The better understanding of those virus–host relationships could lead to new treatment strategies. Indeed, compounds targeting cellular proteins normally hijacked by the virus for its viral cycle could impair its replication. In addition, a comprehensive picture of interactions along the viral cycle could help choose compounds targeting different steps of the viral cycle to avoid the development of resistance to treatment by the virus. It is important to note that many interactions studies, between EIAV and host factors, were not conducted in equine cells, meaning that some species-specific factors—equine—could be missing pieces in the described mechanisms, even though there is high similarity among mammalians proteins. The study of EIAV is a challenge on its own to provide therapeutics to veterinary medicine as well as a promising model to study HIV-1 functioning from another point of view.

## Figures and Tables

**Table 1:** All known described EIAV cellular partners. ELR1: equine *lentivirus* receptor 1; TRIM5α: tripartite motif 5 alpha; CA: capsid; Ref1: restriction factor 1; LV1: lentivirus susceptibility factor 1; SAMHD1: sterile alpha motif and histidine/aspartic domain-containing protein 1; NUP: nucleoporin; NPC: nuclear pore complex; TNPO3: transportin-3; LEDGF: lens epithelium-derived growth factor; TAK: tat-associated kinase; TAR: tat-responsive element; P-TEFb: positive transcription elongation factor; SR proteins: serine arginine proteins; ASF1: alternative splicing factor 1; SF2: splicing factor 2; REBP: Rev/Rex effector binding protein; RRE: rev responsive element; Viperin: virus inhibitory protein endoplasmic reticulum-associated interferon inducible; APOBEC3: apolipoprotein B mRNA editing enzyme-catalytic polypeptide 3; AP-2: AIP1/ALIX: ALG-2 [apoptosis-linked-gene-2 product]-interacting protein X; SNARE: soluble N-ethylmaleimide-sensitive factor attachment protein receptor; Keap1: Kelch-like ECH associated protein 1; Nrf2: nuclear factor erythroid 2-related factor; AREs: antioxidant response elements.

	Host Factor	Interacting Viral Protein	Mechanism(s)	References
1	ELR1	gp90	Binding to the receptor triggers conformational changes and fusion of viral envelope to plasma membrane	[15,27,28,29]
2	TRIM5α	CA	Binds to assembled capsid and prevents uncoating, acts before integration	[33,34,35,36]
3	Ref1/Lv1	CA	Blocks nuclear import of viral DNA, after reverse transcription but before integration	[37,38,39,40]
4	SAMHD1	/	Depletes the nucleotide pool, interfering with reverse transcription	[43,44]
5	SAMHD1/BECN1/PIK3C3	Rev	Rev hijacks BECN1 and PIK3C3 to mediate the degradation of SAMHD1 through lysosomal pathway	[45]
6	equine MX2	CA	Distributed around the nucleus, MX2 binds the capsid and most likely blocks nuclear import of proviral DNA	[41,42]
7	NUP153	CA	Nuclear import of pre-integration complex through the NPC	[53]
8	TNPO3	CA	Binds CA for nuclear import	[54,55,56]
9	LEDGF/p75	Integrase	Binds integrase to guide EIAV lentiviral DNA integration in active transcription sites	[57,58,59]
10	TAK	Tat	Required for viral transcription	[60]
11	equine cyclin T1	Tat	Binding of tat to TAR for EIAV transcription transactivation	[61,62,63]
12	P-TEFb	Tat	Binds tat and TAR and allows transcription of EIAV	[63]
13	PU.1	Tat	Macrophage-specific transcription factor	[63]
14	TRIM32	Tat	Binds to tat activation domain	[64]
15	SR proteins	/	Splicing factors	[65,66]
16	ASF1/SF2	/	Splicing factors	[67,68]
17	REBP	Rev	Mediates nuclear export function of rev	[69]
18	NUP98	Rev	Nuclear export of RRE RNAs	[70]
19	Viperin	Gag, Env, ELR1,	In the endoplasmic reticulum (i) inhibits gag and env budding, (ii) reduces synthesis of env and ELR1, (iii) impairs trafficking of ELR1	[71]
20	SLFN11	/	Impairs viral mRNA translation by interacting with tRNAs	[73,74]
21	ZCCHC3	Gag NC and LTRs	Packaged into virions, inhibits viral RNA packaging and virion production	[75]
22	APOBEC3	NC	Induction of hypermutation of viral cDNA	[46,47,50,51,52]
23	tetherin	Env	Anchors virions at the plasma membrane	[76,77,78,79,80]
24	AP-2		Virion assembly and release	[81]
25	AIP1/ALIX	P9	Virion assembly and release	[82,84,85,86]
26	SNARE	Gag	Virion assembly and release	[88]
27	SERINC3/5	S2	S2 counteracts SERINC5 antiviral activity by excluding it from viral particles	[89,90,91]
28	Keap1	Rev	Rev binds competitively to Keap1 instead of Nrf2, having two antiviral consequences: rev is trapped in the cytoplasm, unable to translocate to the nucleus, and Nrf2 translocates to the nucleus and promotes the transcription of ARE genes which are antiviral	[92]
29	OS9	S2	Unknown	[93]
30	PSMC3	S2	Unknown	[93]
